# 

**Published:** 1891-01

**Authors:** 


### CONTENTS—JANUARY, 1891.—VOL. VI., NO. 7.

ORIGINAL ARTICLES— PAGE.

Five Resections of the Alimentary Canal. B. E. Hadra, M. D .......... 275

A Discussion on Abortion in the Chicago Medical Legal Society .......... 283

Abstract of Closing Remarks in his Address Before the Association; V. A. Howeth, M. D .......... 288

Aneurism of Palmer Arch of Left Hand. R. Menger, M. D .......... 297

Asepsis vs. Antisepsis in the Treatment of Accidental Injuries. B. F. Church, M. D .......... 299

SOCIETY NOTES—

Death of Dr. Underhill .......... 301

Austin District Medical Society .......... 303

Hill County Medical Association, etc .......... 304

ITEMS OF INTEREST—

Notes on Iron, etc .......... 305

EDITORIAL—

Small-pox in Texas.—The Necessity of a State Board of Health Demonstrated .......... 313

Asylum Superintendents Appointed .......... 316

MEDICAL NEWS AND MISCELLANY—

Embracing many items of interest .......... 317

BOOK NOTICES—

Review of several books .......... 320

PUBLISHER'S NOTES—

Containing Valuable Information .......... 321

